# Plasmon-Enhanced Photo-Luminescence Emission in Hybrid Metal–Perovskite Nanowires

**DOI:** 10.3390/nano15080608

**Published:** 2025-04-15

**Authors:** Tintu Kuriakose, Hao Sha, Qingyu Wang, Gokhan Topcu, Xavier Romain, Shengfu Yang, Robert A. Taylor

**Affiliations:** 1 Department of Physics, University of Oxford, Oxford OX1 3PU, UK; 2 School of Chemistry, University of Leicester, Leicester LE1 7RH, UK; 3 Optoelectronics Research Centre, University of Southampton, Southampton SO17 1BJ, UK

**Keywords:** helium droplets, plasmonics, perovskites nanowires, nanophotonics, micro-photoluminescence, time-resolved spectroscopy

## Abstract

Semiconductor photonic nanowires are critical components for nanoscale light manipulation in integrated photonic and electronic devices. Optimizing their optical performance requires enhanced photon conversion efficiency, for which a promising solution is to combine semiconductors with noble metals, using the surface plasmon resonance of noble metals to enhance the photon absorption efficiency. Here, we report plasmon-enhanced light emission in a hybrid nanowire device composed of perovskite semiconductor nanowires and silver nanoparticles formed using superfluid helium droplets. A cesium lead halide perovskite-based four-layer structure (CsPbBr_3_/PMMA/Ag/Si) effectively reduces the metal’s plasmonic losses while ensuring efficient surface plasmon–photon coupling at moderate power. Microphotoluminescence and time-resolved spectroscopy techniques are used to investigate the optical properties and emission dynamics of carriers and excitons within the hybrid device. Our results demonstrate an intensity enhancement factor of 29 compared with pure semiconductor structures at 4 K, along with enhanced carrier recombination dynamics due to plasmonic interactions between silver nanoparticles and perovskite nanowires. This work advances existing approaches for exciting photonic nanowires at low photon densities, with potential applications in optimizing single-photon excitations and emissions for quantum information processing.

## 1. Introduction

In the rapidly advancing field of quantum technology, photonic nanowires have become essential components, driving groundbreaking technological innovations in light manipulation at a nanoscale. These one-dimensional entities, typically made from semiconductor materials, play critical roles in light generation, propagation, detection, and amplification. Their seamless integration with electronic systems positions them as indispensable for the development of next-generation integrated photonic and electronic devices. Their high aspect ratio, coupled with their ability to confine and guide light, makes them particularly promising for applications such as waveguides, photodetectors and light emitting devices [1,2,3]. Moreover, the optical and electronic properties of semiconductor nanowires can be tuned by controlling the synthesis process [4]. In optically pumped nanolaser devices, nanowires can serve as both the gain medium and optical cavity, creating ultra-compact and coherent light sources [5]. Their large surface-to-volume ratio also makes nanowires sensitive transducers for chemical and biological sensing and imaging applications [6,7].

Among the various photonic nanowire materials, perovskite nanowires, particularly those made from cesium lead bromide (CsPbBr_3_), have attracted significant interest in recent years. These nanowires exhibit a strong photoluminescence, narrow emission bandwidth, high charge carrier mobility, and easy solution-phase synthesis [8]. Their ability to lase at low thresholds with high quality factors makes them promising candidates for next-generation nanophotonic light sources [9,10]. The optical properties of CsPbBr_3_ nanowires can be tuned across the visible spectrum through halide compositional engineering [11]. Furthermore, their all-inorganic composition provides improved stability when compared with hybrid organic–inorganic perovskites [12]. Recent advances in controlled synthesis have further allowed for precise tuning of the dimensions and morphology [13]. These mark CsPbBr_3_ nanowires as an emerging material platform for next-generation nanophotonic and optoelectronic devices.

A key challenge in photonic nanowire research is optimizing their optical performance, particularly in terms of the photon conversion efficiency. Recent advancements suggest that combining semiconductor nanowires with noble metals holds great potential for enhancing light emission [14,15]. Plasmonic structures generate strongly confined electromagnetic fields near their surfaces through surface plasmon resonances, enhancing both the absorption and emission processes in nearby semiconductors, which substantially increases the photoluminescence (PL) yield. Notably, a PL enhancement factor of 40 has been observed in Au/CdS core–shell nanoparticles compared with pure CdS particles [16]. Additionally, a 23-fold increase in PL intensity has been reported in CdSe quantum dot nanocrystals [17]. Moreover, metal–semiconductor core–shell nanowires have been predicted to provide a high optical gain for lasing and may be suitable for electrical injection [18]—a critical advancement toward incorporating nanowire lasers into on-chip nanophotonic devices [5]. Despite these promising capabilities, fabricating hybrid metal–semiconductor nanowires with optimal optical properties remains challenging due to the inherent non-wetting behavior between these dissimilar materials.

In this work, we propose a hybrid device based on metal–semiconductor nanowires that can significantly enhance light–matter interactions, indicating that plasmonic enhancement techniques are applicable to perovskite nanowires as well as to quantum dot-based systems. We design a four-layer structure based on CsPbBr_3_, in which the photon absorption of the semiconductor nanowires is enhanced using plasmonic nanoparticles. The insulating layer helps minimize losses by preventing direct charge transfer while still allowing the enhanced electromagnetic field to interact with the semiconductor. To achieve this, we employ an innovative two-phase fabrication approach that combines superfluid helium droplets with wet-chemical synthesis. Helium droplets allow for exceptional control over particle size and, consequently, the plasmonic resonance frequency. This enables us to tune the resonance to complement the semiconductor absorption band. We expect this hybrid architecture to demonstrate significantly enhanced light–matter interactions, potentially at the single-photon level. Through micro-photoluminescence and time-resolved spectroscopy techniques at low temperatures, we characterize these novel nanodevices and establish their potential for quantum information processing and integrated nanophotonic applications.

## 2. Design and Fabrication of a Metal–Perovskite Nanowire Device

**Figure 1:**
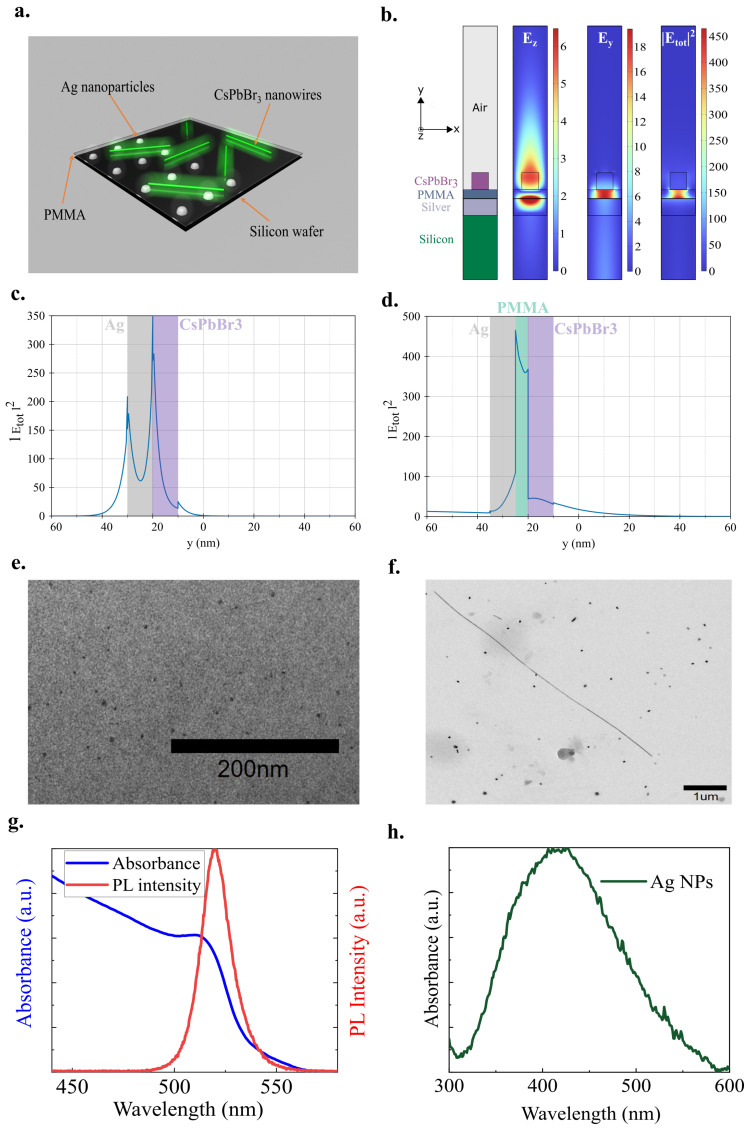
(**a**). Artistic view of the hybrid metal–perovskite nanowires. (**b**). Left: Simplified 2D model for the four-layer structure. Right: Three panels showing the corresponding spatial distribution for $E_{z}$, $E_{y}$ and $|E_{\mathrm{tot}}{|}^{2}$ for the hybridised plasmonic eigenmode at 520 nm. (**c**,**d**). Electric intensity $|E_{\mathrm{tot}}{|}^{2}$ along the vertical *y*-axis without and with the PMMA interlayer, respectively. (**e**). Transmission electron microscopy (TEM) image of silver nanoparticles synthesized by helium droplets. (**f**). TEM images of CsPbBr_3_ nanowires after centrifugation. (**g**). UV-visible absorption and PL spectra of CsPbBr_3_ nanowires. (**h**). UV-visible absorption spectrum of the silver nanoparticles.

Our device is a cesium lead halide perovskite nanowire-based four-layer structure (CsPbBr_3_/PMMA/Ag/Si) of the type illustrated in Figure 1a. It incorporates silver nanoparticles (AgNPs) to activate the plasmonic effect. A dielectric layer has also been placed between the perovskites and AgNPs to reduce plasmon propagation losses while ensuring strong polariton coupling. We highlight the advantages of using a dielectric interlayer by performing finite-element method simulations with COMSOL Multiphysics 6.2. Figure 1b illustrates the working principle using a simplified 2D geometry depicted on the left. The CsPbBr_3_ nanowire is modeled by a 10 nm × 10 nm infinitely long wire, while the plasmonic layer is designed with a 10 nm thin layer of silver. A 5 nm thin PMMA layer separates the nanowire and the plasmonic layer and the whole structure is placed on a silicon substrate. The three additional panels display the spatial distributions of the electric fields $E_{z}$, $E_{y}$, and the total electric intensity $|E_{\mathrm{tot}}{|}^{2}$, respectively, for the hybridized plasmonic eigenmode around 523 nm. The $E_{z}$ field distribution clearly shows the excitation of the plasmonic mode at the silver surface and the photonic mode within the perovskite nanowire. In contrast, the $E_{y}$ field distribution reveals the hybridization of the plasmonic mode and the perovskite mode occurring in the PMMA interlayer. Consequently, the electric intensity $|E_{\mathrm{tot}}{|}^{2}$ is strongly confined within the PMMA, which mitigates the plasmonic quenching that typically negatively affects photoluminescence. Figure 1c,d further illustrate the advantages of employing a dielectric interlayer. Figure 1c shows the enhanced $|E_{\mathrm{tot}}{|}^{2}$ along the *y*-axis for a plasmonic eigenmode without the PMMA interlayer. We observe that most of the electric field enhancement occurs at both silver interfaces, which promotes quenching through non-radiative plasmonic channels. In contrast, Figure 1d demonstrates an even greater field enhancement when the PMMA interlayer is present, with strong field localization within the PMMA, as previously shown in the right panel of Figure 1b.

The fabrication of hybrid metal–perovskite nanowire devices involved several steps. First, plasmonic nanoparticles were formed in superfluid helium droplets and were deposited onto an oxidized silicon wafer (described in more detail below). This method offers significant advantages over traditional nanoparticle synthesis techniques, such as solution-based or gas-phase methods. Specifically, it eliminates the need for surfactants or stabilizers that can contaminate nanoparticle surfaces. Operating under ultra-high vacuum and extremely low temperatures (0.37 K), superfluid helium acts as an inert medium that provides precise control over nanoparticle size and prevents aggregation [19]. This approach allows for the production of nanoparticles with controlled size, high purity, and excellent dispersibility, making it ideal for contamination-free synthesis. Additionally, helium droplets facilitate soft landing of nanoparticles onto substrates without introducing impurities.

In the helium droplet method, helium droplets are formed through the supersonic expansion of pre-cooled high-pressure helium gas (20 bar) through a pinhole nozzle. When passing through the pickup region, these droplets function as very cold nano-reactors, capturing vapor-phase atoms or molecules, which are instantly cooled and subsequently aggregate into nanoparticles building on atoms one by one. In the ultra-high vacuum environment no impurities will be co-added to the helium droplets, ensuring that pure nanoparticles remain after helium evaporation. By controlling the helium droplet size (by nozzle temperature and stagnation pressure), we can control the quantity of captured atoms, thereby precisely tuning nanoparticle sizes. This approach effectively addresses a common challenge in gas-phase synthesis nanoparticle aggregation by isolating individual particles within separate helium droplets, resulting in excellent dispersion of the plasmonic nanoparticles. The method also allows for versatile deposition onto various substrates. Transmission electron microscopy (TEM) images obtained with a JEOL JEM-1400 TEM microscope (Japan) (see Figure 1e) demonstrate the successful deposition of high-purity silver nanoparticles with minimal clumping on oxidized silicon substrates with an areal density of $6\times 10^{11}$ cm^−2^. As illustrated in Figure 1g, the integration of AgNPs and perovskite nanowires has resulted in photoluminescence that peaks at ~520 nm, matching well with the plasmonic absorbance seen for our bare Ag nanoparticles in Figure 1h.

A polymethyl methacrylate (PMMA) layer was deposited using a spin coater after the deposition of plasmonic nanoparticles. PMMA was chosen as the dielectric layer due to its favorable properties for controlled fabrication and its compatibility with various deposition techniques. It offers excellent optical transparency and stability, making it suitable for plasmonic applications where the dielectric layer is crucial in modulating light–matter interactions. Furthermore, PMMA is easy to process, allowing for precise control over film thickness, which is essential when optimizing the device performance [20,21]. Supplementary Figure S2 shows the thickness of the PMMA thin film at different concentrations (refer to Section S1 of the Supplementary Information for fabrication details).

To synthesize the perovskite nanowires, we employed solution processing (refer to Section S1 of the Supplementary Information for fabrication details), followed by spin-coating onto a silicon substrate that was coated with PMMA and silver nanoparticles. As shown in Figure 1f, the fabricated nanowires have an approximate diameter of 30 nm and a length of ∼7 μm. Figure 1g shows the absorption spectra and photoluminescence emission of the perovskite nanowires. The CsPbBr_3_ nanowires exhibit absorption and emission peaks at 514 nm and 520 nm, respectively.

## 3. Optical Characterization

**Figure 2:**
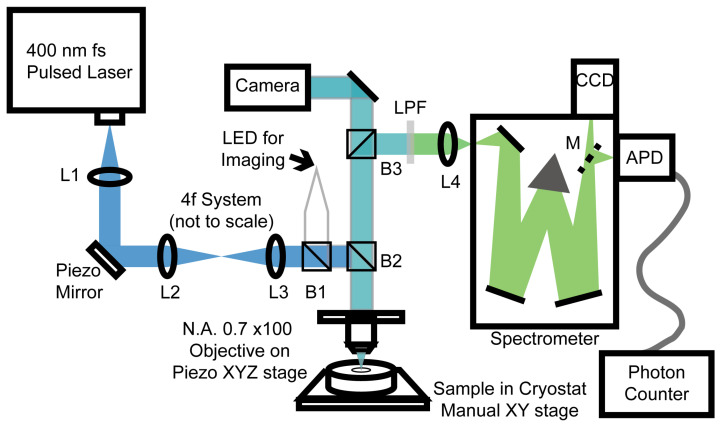
A schematic diagram of the confocal micro-photoluminescence (PL) setup shows a femtosecond laser emitting light at 400 nm, focused onto a sample using a 100× microscope objective. The sample is positioned on an xy translation stage within a cryostat for low-temperature measurements. The photoluminescence spectrum is analyzed by a charge-coupled device and an avalanche photodiode with photon-counting electronics at the spectrometer’s exit slit.

We investigated the optical properties of hybrid metal–perovskite nanowires (NWs) using micro-PL spectroscopy. The setup is illustrated in Figure 2. We illuminated the metal–perovskite NWs with an above-bandgap excitation wavelength of 400 nm, using a 100 fs frequency-doubled Ti: Sapphire laser (Coherent Mira) operating at a repetition rate of 76 MHz. The incident laser power on the hybrid device varied from a few nanowatts (nW) to a few microwatts (μW). The sample was mounted in a Janis continuous flow liquid helium cryostat, allowing us to conduct experiments at temperatures near 4 K. These low-temperature measurements enabled us to resolve any fine structural characteristics of the sample. A 100× magnification Mitutoyo microscope objective with a numerical aperture of 0.7 was used to focus the incident laser beam to a spot size of approximately 1 μm. The emitted PL was directed to an Andor Shamrock 0.3 m grating spectrometer equipped with an Andor electron multiplying charge-coupled device for spectral characterization. A telecentric 4f scanning system was also employed for micro-PL 2D mapping, which allowed us to vary the incident angle of the exciting laser at the entrance to the objective using a piezoelectric mirror. This provided an independent method for moving the excitation spot on the sample relative to the collected emission, which was imaged confocally through the center of the objective. Time-resolved photoluminescence (TRPL) measurements were performed using the same experimental setup, with the emitted PL directed to an MPD Si avalanche photodiode connected to a Picoquant time-correlated photon counting system with a time resolution of 50 ps.

**Figure 3:**
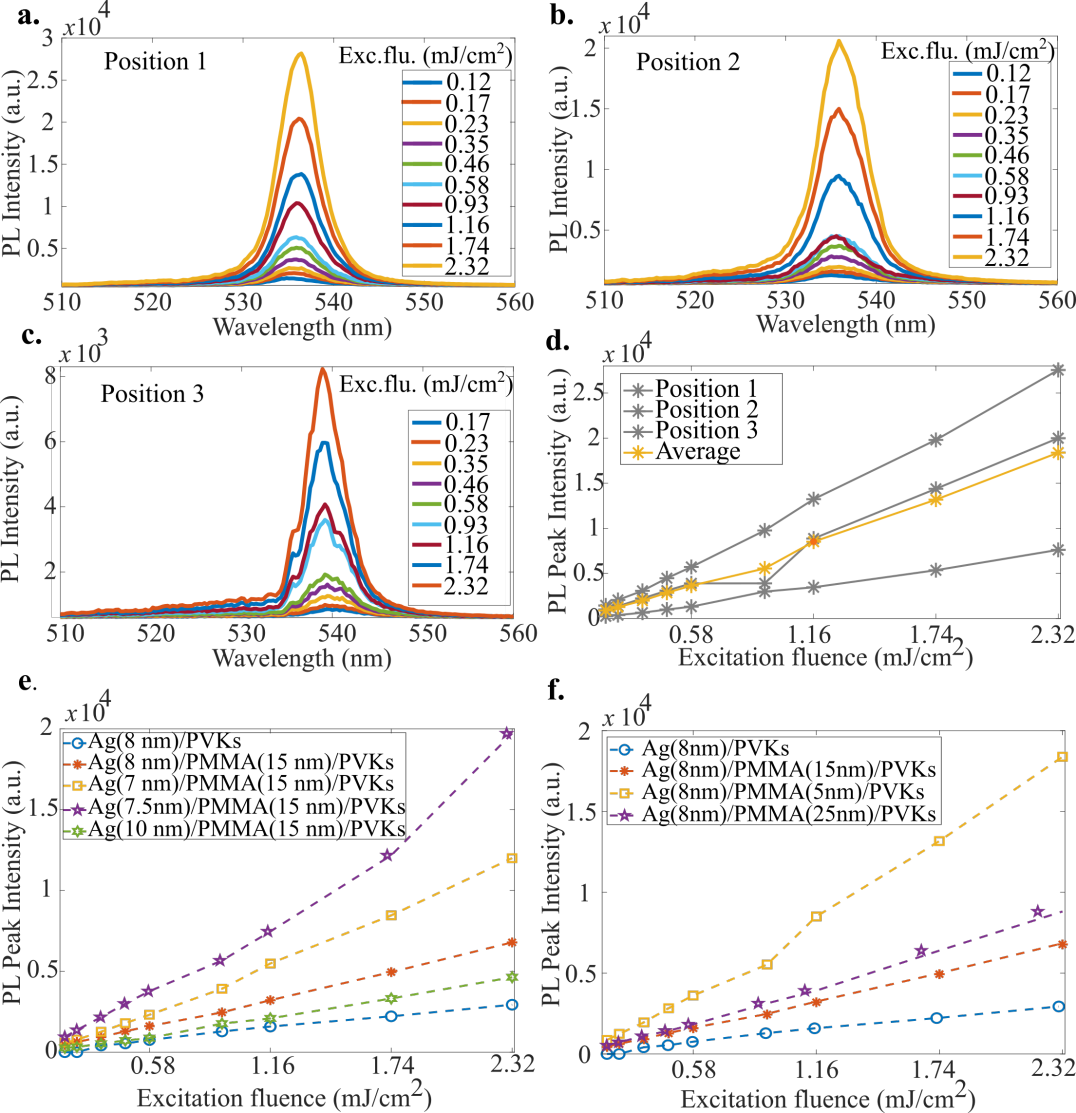
(**a**–**c**). Power-dependent micro-PL spectra taken at three different positions on the hybrid device. At position 3, the individual emission lines form several single nanowires that can be resolved in the spectra. (**d**). Position-dependent PL peak intensity as a function of excitation fluence on the hybrid device. (**e**,**f**). Optimization of device parameters by varying the AgNPs size and PMMA layer thickness.

We first conducted excitation power-dependent PL measurements at various locations on the hybrid metal–perovskite NWs, as shown in Figure 3a–c. The measurements at 4 K reveal a bright and narrow PL feature with a full width at half-maximum (FWHM) of 1.3 nm, which is likely to be the PL emission behavior of a single NW coupled with the Ag particle. It is noteworthy that by using a sample stage capable of adjusting the laser beam on a micrometer scale, similar peaks were easily identified throughout the sample. However, in most cases, the PL peaks exhibited collective emission from multiple NWs, resulting in an FWHM of 6–7 nm. The peak intensities varied due to the coupling of different nanostructures, but the integral over a specific range, where the average number of NWs could reasonably represent the PL intensity of the structure of interest, remained consistent. The details of the integral mapping will be discussed later. To accurately determine the emitted PL intensity from the device, position-dependent PL measurements were taken, followed by the estimation of the average PL peak intensity as a function of excitation fluence, as shown in Figure 3d.

To optimize the device parameters, a series of samples with varying AgNPs sizes and dielectric layer (PMMA) thicknesses were investigated, using a similar averaging method, but focusing only on those peaks precisely located at optimal coupling sites where the peaks exhibited relatively narrow FWHM. Figure 3e,f compares the PL intensities for different device parameters. The comparison indicates that AgNPs with a diameter of 7.5 nm and a dielectric layer thickness of 5 nm yielded the best performance in the PL emission. Therefore, we selected these parameters as the optimal configuration. It is worth noting that the metallic particles produced by superfluid helium droplet vortices and directly projected onto the Si substrate exhibited some size distribution. However, over 50% of the Ag nanoparticles were within the designed size of 7.5±1 nm. The control over the thickness of the spin-coated dielectric layer and the specific size distribution of the metal particles is detailed in the supplementary information. Additionally, we repeated the measurements over several days, ensuring that the samples were consistently kept in a dry nitrogen atmosphere or under vacuum in the cryostat, as it is well-known that pure perovskite nanowires are susceptible to moisture, we observed no signs of degradation.

**Figure 4:**
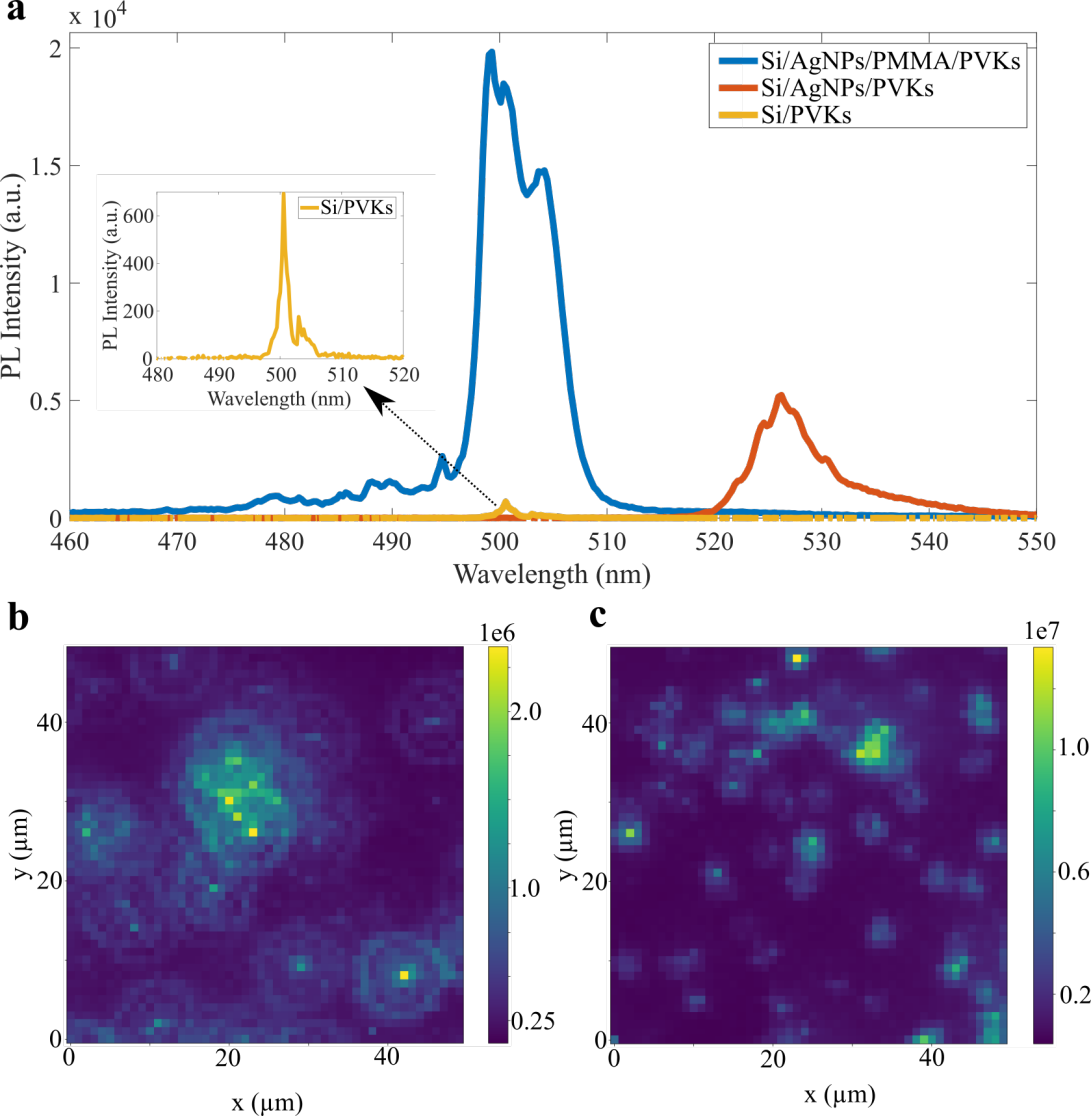
(**a**) PL emission spectra from various device configurations. (**b**) 2D mapping on pure perovskite nanowires. (**c**) 2D mapping on hybrid metal-perovskite nanowires.

After modifying the device parameters, a comprehensive investigation of the device was conducted. Figure 4a presents PL spectra for pure perovskites and hybrid devices with and without a buffer layer at 4 K. A PL enhancement of 29 times was observed in the hybrid metal–perovskite NW device compared to the pure perovskite NWs. This large enhancement is attributed to the introduction of the dielectric layer, which acts as a spacer to modify the field distribution, preventing excessive absorption of incident light by the Ag particles and ensuring sufficient photon–plasmon coupling, as confirmed both by this study and previous studies [22,23,24] where SiO2 was used as a spacer. Furthermore, an enhancement of approximately 7.5 times was observed with the presence of AgNPs compared to bare perovskite NWs, which is consistent with previous studies, demonstrating the coupling of plasmons with the PL emission from perovskites [25,26,27].

Although a strong plasmonic enhancement in PL emission was observed, the collective emission from hybrid NWs remained an issue. To address this, a series of 2D scans were conducted in a specific area where the averaged emission could be observed. This was done to determine the average enhancement arising from the fact that both the distribution of Ag particle and nanowires is random. Figure 4b,c presents the mapping of pure perovskite NWs and hybrid NWs with optimized parameters. The scan covered an area of 50×50 μm, with each pixel representing 1×1 μm. We repeated scans of this kind over different parts of both samples, and an average enhancement of 8.5±2.4 was observed. Preliminary measurements (See Supplementary Figure S4) taken at room temperature show an enhancement factor of up to 9.1; however, here the emission is thermally broadened and shifted to lower energy, and the overlap with the Ag plasmonic mode is, therefore, slightly reduced.

**Figure 5:**
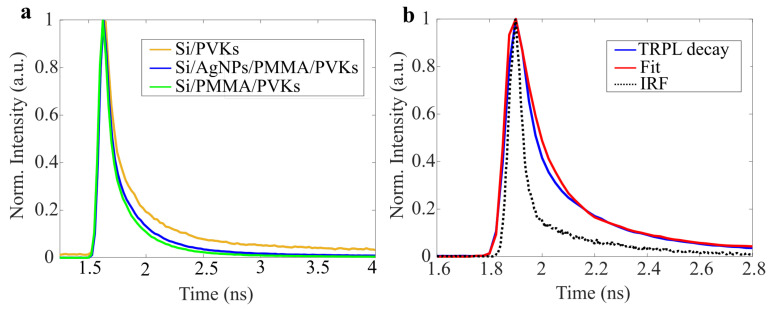
(**a**) Comparison of time-resolved photoluminescence for various device configurations. (**b**) An example of extracting lifetime values using the exponential deconvolution method on Si/AgNPs/PMMA/PVKs.

Lifetime measurement is another crucial aspect that can further verify that the enhanced emission is due to plasmonic coupling, as the presence of localized surface plasmons effectively increases the radiative decay rate, resulting in a shorter lifetime. However, given the configuration of the hybrid NWs, there could also be an extended lifetime due to the surface states of the semiconductor NWs, which form traps in the PL emission process. While the radiative decay is enhanced, the reduced non-radiative recombination could subsequently extend the lifetime. A more detailed study based on the structure is needed to fully understand the emission dynamics; however, the extreme brightness seen points to the dominance of radiative processes here. In Figure 5a, we present time-resolved PL measurements of the corresponding samples. In Figure 5b, the experimental results are fitted with exponential decays to extract lifetime values, thereby verifying a small effect on the recombination dynamics arising from plasmon-enhanced local fields. The obtained lifetime values were 71 ps, 64 ps, and 61 ps for Si/PVKs, Si/AgNPs/PMMA/PVKs, and Si/PMMA/PVKs, respectively. Introducing Ag results in a slight decrease in the lifetime and a clear suppression of the longer lived tail seen in the recombination dynamics of the bare perovskite nanowires on the Si substrate alone. However, due to the resolution limits of the instrument, the observed difference in the fast decay is insufficient to make any definitive statements about the true magnitude of the lifetime reduction due to plasmonic coupling. Therefore, further research into the lifetime measurements is required.

## 4. Conclusions

In this work, we demonstrate significant plasmon-enhanced light emission from a hybrid device composed of silver nanoparticles and cesium lead halide perovskite nanowires. We developed a two-phase fabrication method that combines superfluid helium droplet technology with wet-chemical synthesis to create a precisely engineered four-layer nanostructure. The use of superfluid helium droplets provides exceptional control over the nanoparticle size distribution and dispersion, enabling precise tuning of plasmonic resonance frequencies to optimally complement the semiconductor absorption bands. The resulting hybrid nanostructures are found to have dramatically improved the photonic properties, with microphotoluminescence measurements revealing up to a 29-fold increase in emission intensity at 4 K compared to conventional semiconductor structures. Time-resolved spectroscopy further confirms the accelerated carrier recombination dynamics resulting from the engineered plasmonic interactions. The exceptional performance of these hybrid nanostructures sheds light on their potential for applications that require efficient light-matter interactions at low excitation powers [28]. This establishes a solid foundation for further development of advanced nanophotonic devices, including lead halide perovskite-based micro and nanolasers operating in the visible range, as well as for developing practical devices such as LEDs and photodetectors, by enhancing light emission and absorption, respectively. The controlled integration of plasmonic and semiconductor components achieved in this work addresses key challenges in nanophotonic device engineering and represents an important advance toward practical on-chip photonic technologies.

## Data Availability

The data presented in the figures in the paper are available to download from the Oxford Research Archive at https://ora.ox.ac.uk/objects/uuid:056bdd01-0714-4ab9-a970-c8d309d47385 accessed on 12 March 2025.

## Supplementary Materials

The following supporting information can be downloaded at: https://www.mdpi.com/article/10.3390/nano15080608/s1, Figure S1. Schematic of ultra-high vacuum helium droplet apparatus for the synthesis of silver nanoparticles. (a) xyz manipulator; (b) nozzle; (c) skimmer; (d) pick up region including a alumina oven with silver inside; (e) deposition station. Figure S2. Obtained PMMA layer thickness with different concentrations. Figure S3. TEM images of CsPbBr_3_ nanowires with different reaction time. a. 15 min; b. 30 min; c. 45 min; d. 60 min. TEM images of CsPbBr_3_ nanowires with different centrifugation times (e to g). Figure S4. Photoluminescence spectrum measured at room temperature for the perovskites only (Si/PVKs) and the hybrid metal-perovskite structures (Si/AgNPs/PMMA/PVKs). References [19,29,30,31,32,33,34,35] are cited in the Supplementary Materials.
