# Supplementary material for: Plasmon-Enhanced Photo-Luminescence Emission in Hybrid Metal–Perovskite Nanowires
Source: Nanomaterials (Basel). 2025 Apr 15;15(8):608. doi: 10.3390/nano15080608 (PMC12029369; doi:10.3390/nano15080608)
Supplement: Supplementary file 1 [file nanomaterials-15-00608-s001.zip › nanomaterials-3555479-supplementary.pdf]

Plasmon enhanced photo-luminescence emission in hybrid metal-perovskite nanowires

S1. FABRICATION OF METAL-PEROVSKITE NANOWIRE DEVICE

A. Synthesis of plasmonic nanoparticles using He droplet technology

Traditional methods for synthesizing nanoparticles include solution-based methods, gas-phase methods, template-based methods, electrochemical methods, etc. [1–3]. Although these techniques are widely used, each has its limitations. For example, many synthesis methods require surfactants or stabilizers, which can contaminate the surface of the nanoparticles [4]. In some cases, achieving precise control over reaction conditions is challenging, leading to inconsistent particle sizes [5]. Furthermore, nanoparticles can aggregate during the synthesis process, compromising their dispersion and activity [6].

In contrast, the helium droplet method offers significant advantages by allowing nanoparticle synthesis at extremely low temperatures in a high-purity environment, all without the need for surfactants or stabilizers that could introduce contamination [7]. The size of the nanoparticles can be precisely controlled by adjusting the size of the helium droplets, which is a distinct advantage over conventional methods. This technique employs the unique properties of superfluid helium (^{4}He) at very low temperatures to synthesize nanoparticles. The superfluid phase occurs below 2.18 K [8], where liquid helium exhibits nearly zero viscosity and extremely high thermal conductivity. A notable feature of superfluid helium is the presence of quantized vortices within the fluid.

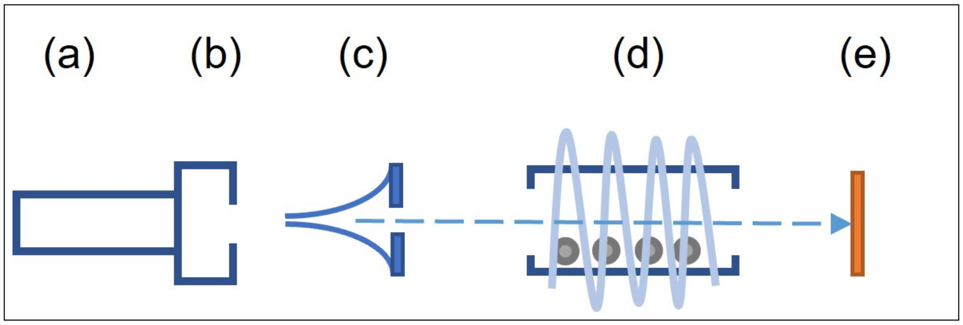

Figure S1. Schematic of ultra-high vacuum helium droplet apparatus for the synthesis of silver nanoparticles. (a) *xyz* manipulator; (b) nozzle; (c) skimmer; (d) pick up region including a alumina oven with silver inside; (e) deposition station.

The apparatus for synthesizing silver nanoparticles using helium droplets is illustrated in Figure S1. It typically consists of a continuous helium droplet source, oven evaporators, and a nanoparticle deposition station. In summary, high-purity helium gas is precooled by a refrigeration compressor and then expanded supersonically through a 5 mm diameter nozzle into a vacuum, forming helium droplets. The stagnation pressure at the nozzle is around 15 bar, and the temperature is precisely controlled at approximately 7.5 K. The nozzle is mounted on an *xyz* manipulator, allowing precise adjustments to its position relative to the skimmer. As the helium droplets pass through the pick-up region, they capture silver atoms generated by evaporating silver in an alumina oven. The concentration of silver vapor can be adjusted by controlling the oven temperature, which allows for fine-tuning of the number of silver atoms captured by the helium droplets. Once the droplets capture the silver atoms and form silver nanoparticles, they move to the next stage and reach the substrate. At this point, the helium droplets facilitate the soft landing of the silver nanoparticles onto the substrate and subsequently evaporate, leaving behind a uniform layer of silver nanoparticles.

B. Deposition of PMMA

To prepare the PMMA solution, 50 mg of PMMA particles were dissolved in 10 ml of toluene and heated at 60 °C for 30 minutes. This solution can be further diluted to achieve PMMA solutions of varying concentrations. The resulting PMMA solution was then applied to the substrate using a spin coater, producing PMMA thin films with different thicknesses, as illustrated in the Figure S2.

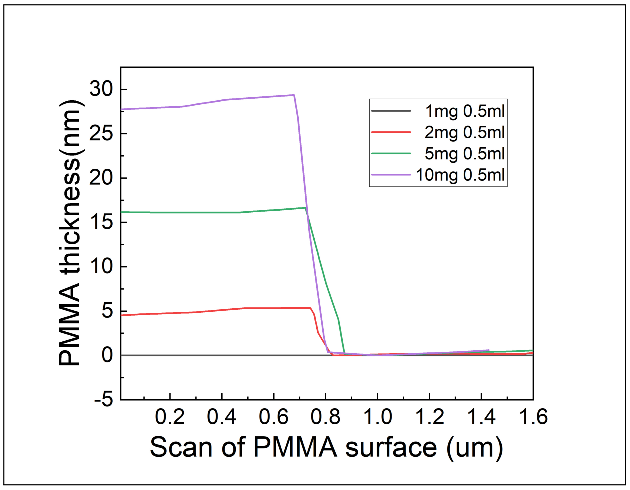

Figure S2. Obtained PMMA layer thickness with different concentrations.

C. Synthesis of perovskite nanowires

Perovskite nanowires were synthesized using a solution-process method. In a typical colloidal synthesis, a mixture of 0.5 ml oleylamine, 0.5 ml oleic acid, and 10 ml 1-octadecene was combined with 0.1 mmol Cs_2CO_3 and 0.3 mmol $PbBr_2$ precursor powders. The mixture was heated to 125 °C for 60 minutes with magnetic stirring. Transmission electron microscopy (TEM) images in Figure S3 a. to d. show the synthesized $CsPbBr_3$ perovskite nanowires with different reaction time. After the reaction, unreacted materials and excess ligands were removed by centrifugation at 6000 rpm for 5 minutes, and the resulting particles were dispersed in 1.5 ml of hexane. The product consisted of both nanowires and a small proportion of nanocubes. To isolate the nanowires, the colloidal solution was centrifuged at 3000 rpm for 5 minutes. The sediment was redispersed in 5 ml of hexane, and the centrifugation-redispersion process was repeated to obtain high-purity $CsPbBr_3$ perovskite nanowires. After the purification, Figure S3 e. to f. show that the obtained nanowires have a diameter of approximately 30 nm and a length of about 7 um.

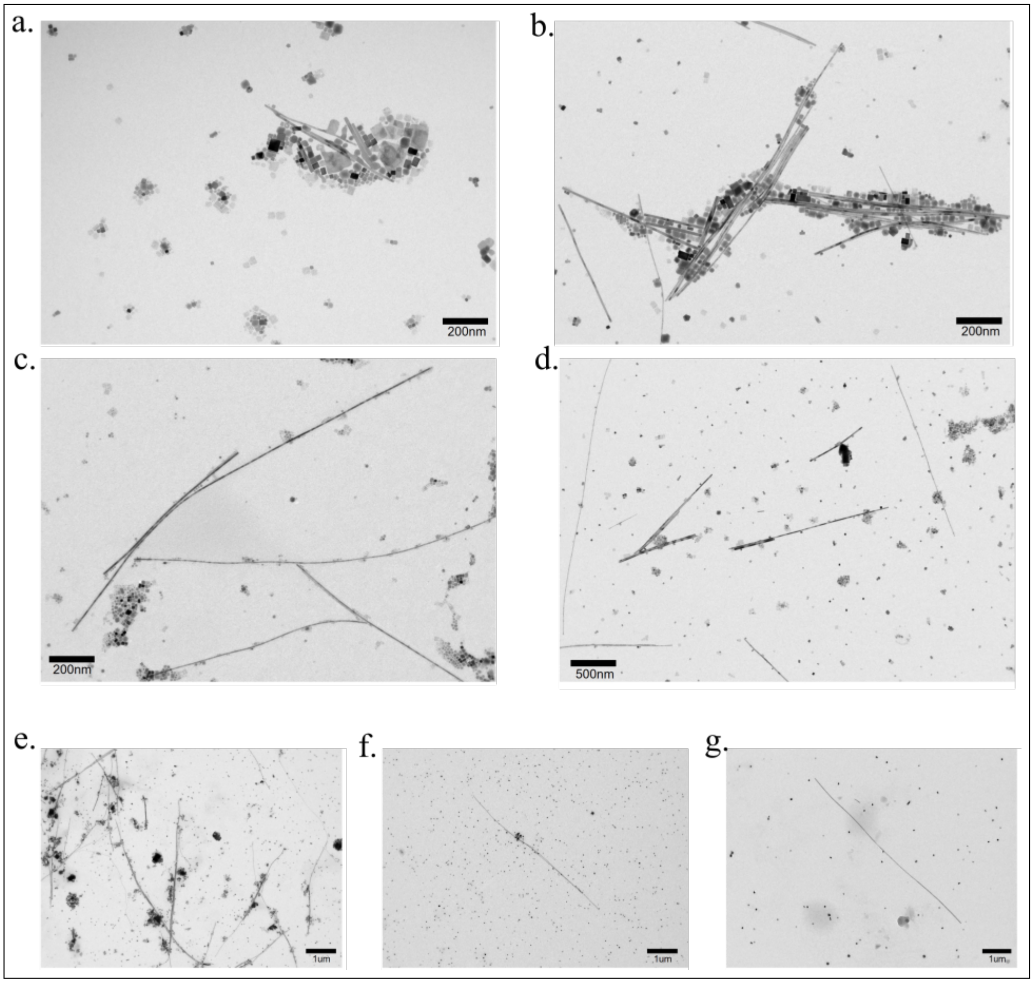

Figure S3. TEM images of CsPbBr3 nanowires with different reaction time. a. 15 min; b. 30 min; c. 45 min; d. 60 min. TEM images of $CsPbBr_3$ nanowires with different centrifugation times (e to g.).

D. Room temperature photoluminescence measurements

Preliminary results of the enhancement of PL at room temperature (RT) for both pure perovskite nanowires and hybrid metal-perovskite nanowires are included in Figure S4. As indicated in the figure, we observed a PL intensity enhancement factor of 9.1 at RT compared to the pure perovskite nanowires.

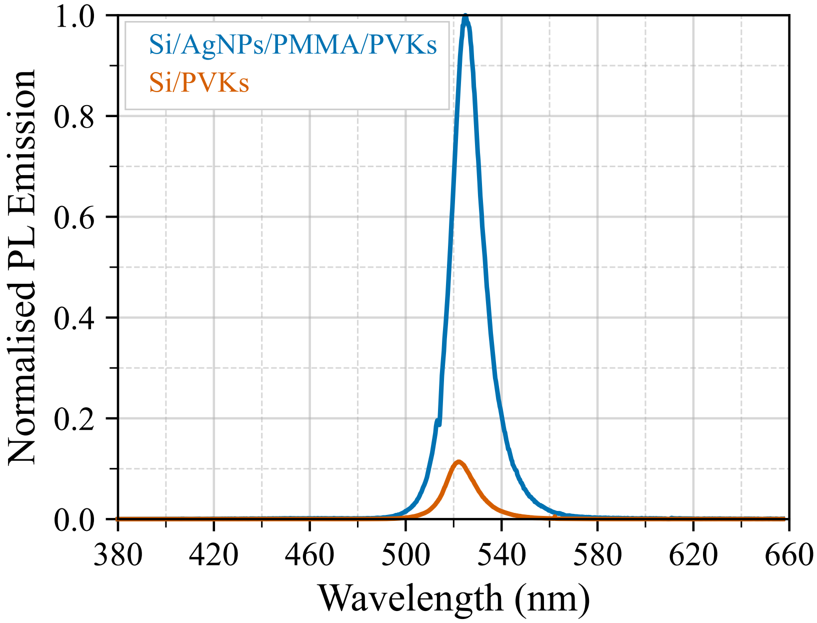

Figure S4. Photoluminescence spectrum measured at room temperature for the perovskites only (Si/PVKs) and the hybrid metal-perovskite structures (Si/AgNPs/PMMA/PVKs).

REFERENCES

1. N. Baig, I. Kammakakam, and W. Falath, "Nanomaterials: a review of synthesis methods, properties, recent progress, and challenges," **2**, 1821–1871. Publisher: Royal Society of Chemistry.
2. P. Grammatikopoulos, T. Bouloumis, and S. Steinhauer, "Gas-phase synthesis of nanoparticles: current application challenges and instrumentation development responses," **25**, 897–912. Publisher: Royal Society of Chemistry.
3. B. Karafinski and N. Sinha, "Current trends in macromolecular synthesis of inorganic nanoparticles," **14**, 862–875.
4. G. Li, D. N. Zakharov, S. Sikder, *et al.*, "In situ monitoring of non-thermal plasma cleaning of surfactant encapsulated nanoparticles," **14**, 290. Number: 3 Publisher: Multidisciplinary Digital Publishing Institute.
5. A. A. Yaqoob, K. Umar, and M. N. M. Ibrahim, "Silver nanoparticles: various methods of synthesis, size affecting factors and their potential applications–a review," **10**, 1369–1378.
6. L. Song, Y. Wang, M. Yang, and Y. Huang, "Investigation on stability of silver nanoparticles with different ligands," **25**, 255.
7. S. Yang, A. M. Ellis, D. Spence, *et al.*, "Growing metal nanoparticles in superfluid helium," **5**, 11545–11553. Publisher: Royal Society of Chemistry.
8. F. Pobell, *Matter and Methods at Low Temperatures* (Springer).
